# Supplementary material for: Local genetic sex differences in quantitative traits
Source: Nat Commun. 2025 Aug 6;16:7232. doi: 10.1038/s41467-025-62504-4 (PMC12328583; doi:10.1038/s41467-025-62504-4)
Supplement: Supplementary file 2 — Description of Additional Supplementary Files [file 41467_2025_62504_MOESM2_ESM.pdf]

Description of Additional Supplementary Files

Supplementary Data 1. Traits

Supplementary Data 2. Error rates in simulation

Supplementary Data 3. Heritability thresholds
